# Supplementary material for: FilamentSensor 2.0: An open-source modular toolbox for 2D/3D cytoskeletal filament tracking
Source: PLoS One. 2023 Feb 6;18(2):e0279336. doi: 10.1371/journal.pone.0279336 (PMC9901806; doi:10.1371/journal.pone.0279336)
Supplement: S1 File — (PDF) [file pone.0279336.s001.pdf]

Supporting information

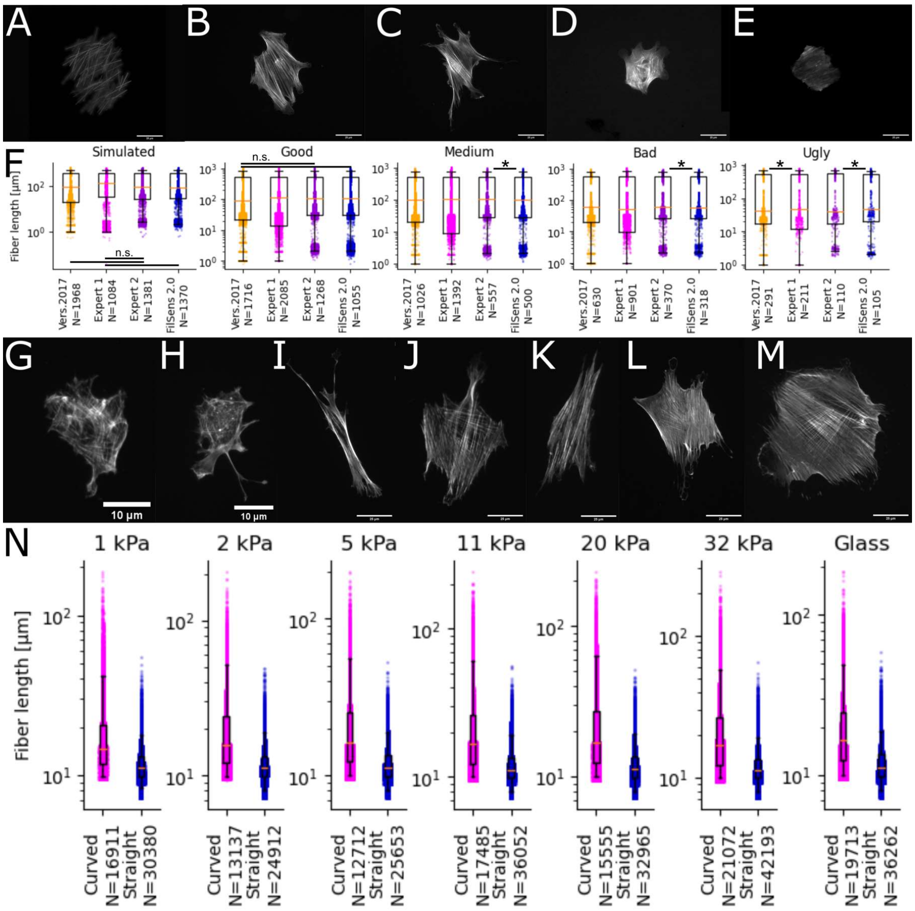

S1 Fig. Old and new ground truth examples. Example pictures and Data for ground truth used in Eltzner 2015 [1] and new ground truth established here. **A-E:** Example images of old ground truth data of different image grades; **A:** simulated, **B:** good, **C:** medium, **D:** bad, **E:** ugly. **F:** Comparison of filaments annotated by FS (2017), Expert 1 (2017), Expert 2 (2021) and FS2.0 (2021), all using straight filaments. **G-M:** Example images of the new ground truth dataset, which contains cells on different substrates; **G:** 1 kPa PA gel, **H:** 2 kPa PA gel, **I:** 5 kPa PA gel, **J:** 11 kPa PA gel, **K:** 20 kPa PA gel, **L:** 32 kPa PA gel, **M:** glass. **N:** Example plot of the new ground truth dataset analyzed with straight and curved filament setting. Number of cells in dataset and number of filaments found indicated on x-axis. We performed a Kolmogorov–Smirnov test between each pair of conditions. n.s. = >0.05, * = <0.05, ** = <0.01, all other conditions <0.001.

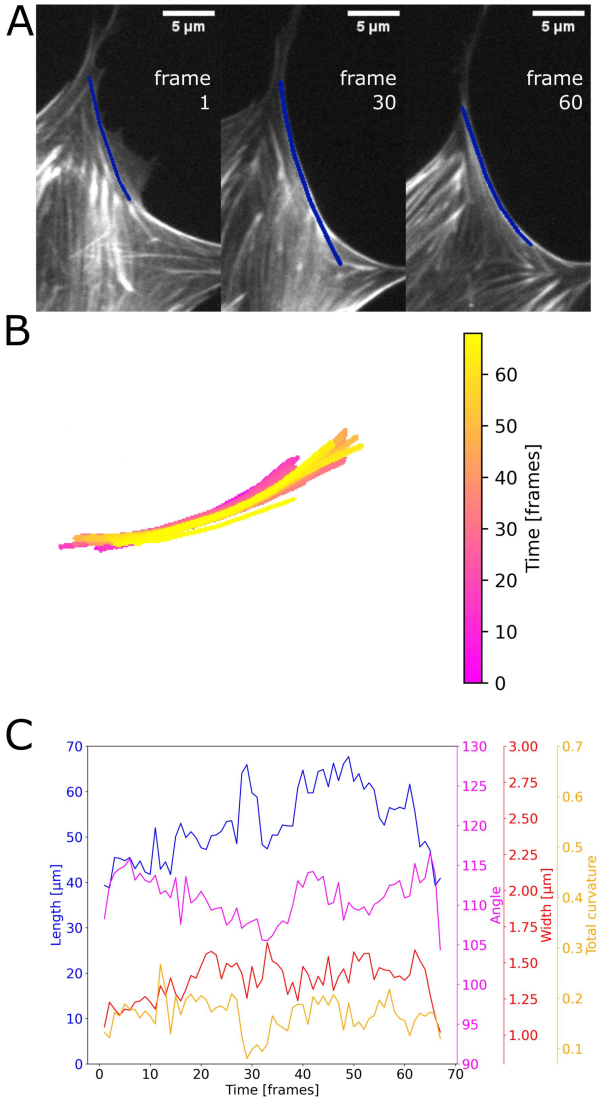

S2 Fig. Exemplary changes of a single filament. Filament shown is the longest filament from cell in Figure 4 with a lifetime of 67 frames. **A:** Example images over time with filament marked. **B:** Overlay of filament over time, color-coded. **C:** Plot of descriptive features of filament over time: Length, angle, width and total curvature.

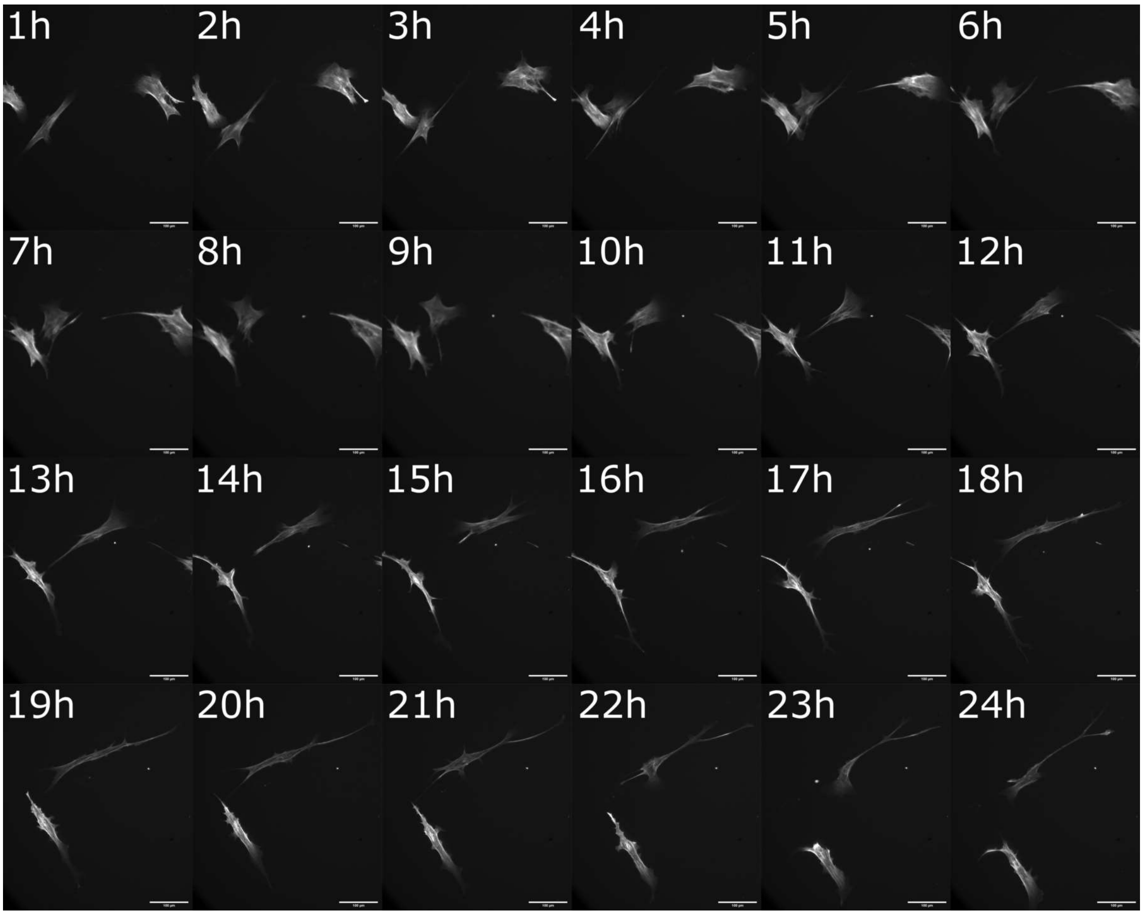

S3 Fig. Example images of the test data used for bounding box feature and speed test. Shown are example images from a 24 h (144 frames) image set used to estimate performance in the FS2.0 and available in the software. As the images contain 3 cells with different brightness which touch and leave the field of view over time, we decided on this image set for general tests of the software during development.

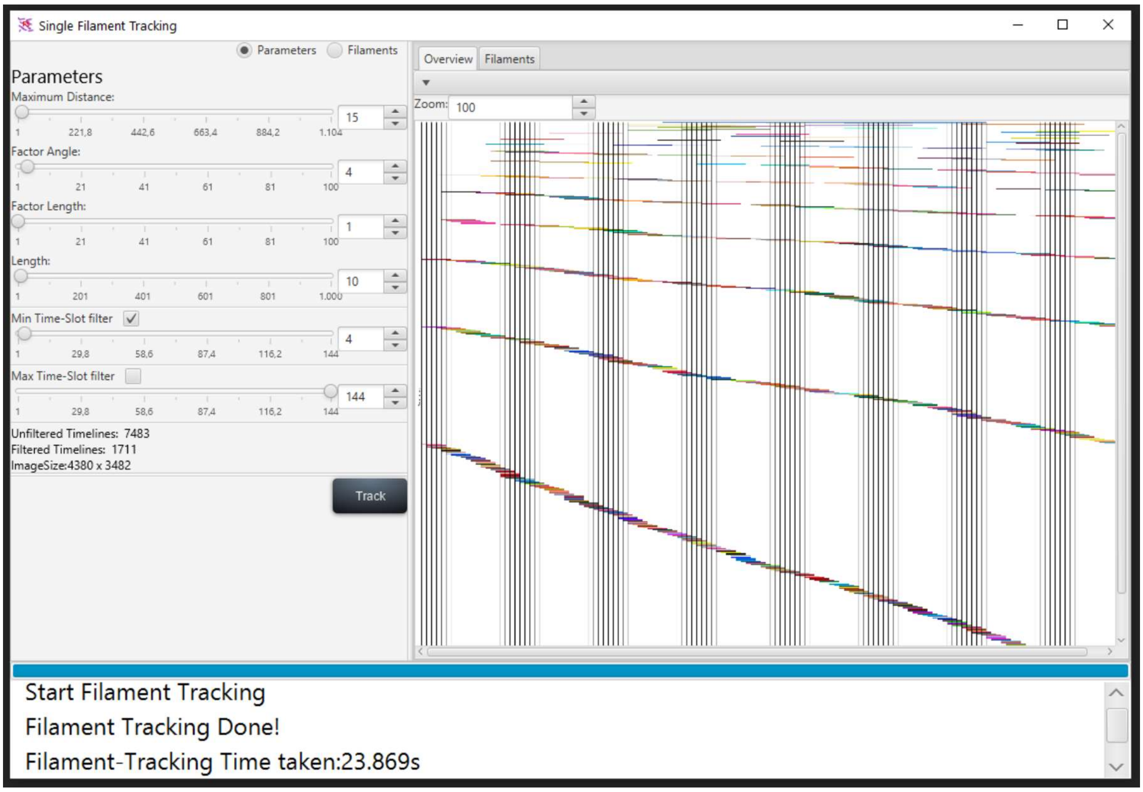

S4 Fig. FS2.0 GUI for Single Filament Tracking. GUI for Single Filament Tracking opens on click on respective button in the filament results page of the main FS2.0 GUI. User has option to set parameters and view and discard filaments. Filament view is given either as timelines or cell overlay. Filaments are color-coded for their lifetimes in frames both in the lifetime view and the filament over cell overlay.

S5 Code

```
%\begin{lstlisting}[frame=single]
package filters;

import ij.process.ImageProcessor;
import javafx.beans.property.*;
import javafx.beans.value.ChangeListener;
import util.Annotations;
import java.io.IOException;
import java.io.ObjectInputStream;
import java.io.ObjectOutputStream;

/**
 * for detailed explanation (transient, writeObject) see FilterCrossCorrelation
 */
//this @Annotations.FilterUI() shows that this class is a filter for generic GUI
//generation the interfaces it implements depend on what kind of filter it is.
//if its for single image or whole stack
//IFilterPrecalc notes that there is some values pre calculated that only depends on
//the params and not on the image.
@Annotations.FilterUI()
public class FilterLaPlace implements IImageFilter, IFilterPrecalc, IFilterObservable {

    public static final int MaskTypeA = 0;
    public static final int MaskTypeB = 1;

    @Annotations.FilterUIField(type = Annotations.FilterUIType.slider, label = "Factor")
    @Annotations.Min(0)
    @Annotations.Max(5)
    @Annotations.Default("core.settings.Pre.lpfac")
    private final transient DoubleProperty factor;
    //this is the property(parameter) for this filter, if implementing a new filter
    //you have to create properties for all parameters you use
    //the annotations above define fields for the GUI (type of field, value range, label...)
    //for different field types check other filters
    //for every property you need getter and setter methods for the \\
    //Serialization (storage as xml) those methods see below
    @Annotations.FilterUIField(type = Annotations.FilterUIType.combobox, label = "Neighborhood")
    @Annotations.Values("filters.supplier.LaPlaceSupplier")//has to be implemented somehow
    @Annotations.Default("core.settings.Pre.lpmask")
    private final transient IntegerProperty maskType;

    private float[] mask = null;

    //the Class needs a default constructor (empty parameter list in constructor)
    public FilterLaPlace() {
        factor = new SimpleDoubleProperty();
        maskType = new SimpleIntegerProperty();
    }
    public FilterLaPlace(int type, double factor) {
```

```
    this();
    setFactor(factor);
    setMaskType(type);
}
//the custom writeObject and readObject are necessary for custom serialization
//(since javaFX properties don't support xml serialization by default)
private void writeObject(ObjectOutputStream s) throws IOException {
    //do that stuff for every property you use in the filter
    //care for the data types (writeDouble for double writeInt for int, etc.)
    s.defaultWriteObject();
    s.writeDouble(getFactor());
    s.writeInt(getMaskType());
}
private void readObject(ObjectInputStream s) throws IOException, ClassNotFoundException {
    //same as in write object
    s.defaultReadObject();
    setFactor(s.readDouble());
    setMaskType(s.readInt());
    // set values in the same order as writeObject()
}
//the above mentioned getter and setter methods (getFactor, factorProperty, setFactor)
public double getFactor() {
    return factor.get();
}
public DoubleProperty factorProperty() {
    return factor;
}
public void setFactor(double factor) {
    this.factor.set(factor);
}
public int getMaskType() {
    return maskType.get();
}
public IntegerProperty maskTypeProperty() {
    return maskType;
}

public void setMaskType(int maskType) {
    this.maskType.set(maskType);
}
//if your filter should force parallel processing return true, otherwise keep \\
//it like that
@Override
public boolean forceParallel() {
    return false;
}
//the actual method that processes the image (filter logic here)
@Override
public void run(ImageProcessor image) {
    if (mask == null) return;
```

```
        image.convolve(mask, 3, 3);
    }

    @Override
    public String toString() {
        return this.getClass().getName() + ":" + getFactor() + "," + getMaskType();
    }
    //if you have stuff to pre calculate, its done here (if your \\
    //filter doesn't need precalc, don't implement
  //the interface and this method can be removed (or just not added)
    @Override
    public void preCalc() {
        double[] mFactors;
        switch (getMaskType()) {
            case MaskTypeA:
                mFactors = new double[]{1, getFactor(), 0};
                break;
            case MaskTypeB:
                mFactors = new double[]{1, 0, getFactor()};
                break;
            default:
                throw new IllegalArgumentException("FilterLaPlace - illegal MaskType");
        }
        float a = (float) -mFactors[2], b = (float) (-mFactors[2] - mFactors[1]),
                c = (float) (mFactors[0] + 4 * mFactors[1] + 8 * mFactors[2]);
        mask = new float[]{a, b, a, b, c, b, a, b, a};
    }
    public float[] getMask() {
        return mask;
    }
    public void setMask(float[] mask) {
        this.mask = mask;
    }
    //these methods are for keeping track of changed parameters, if \\
    //preview is active, the filter will be
    //executed if the parameters are changed
    @Override
    public void addListener(ChangeListener<Object> listener) {
        factorProperty().addListener(listener);
        maskTypeProperty().addListener(listener);
    }

    @Override
    public void removeListener(ChangeListener<Object> listener) {
        factorProperty().removeListener(listener);
        maskTypeProperty().removeListener(listener);
    }
}
```

Other changes neccessary:
in GUI sources, package model, class MainModel in the constructor add your filters in the

following part:

- `clsApplicableFilters = FXCollections.observableArrayList()`
- `clsApplicableFilters.add(FilterGauss.class.getName())`
- `clsApplicableFilters.add(FilterLaPlace.class.getName())`
- `clsApplicableFilters.add(FilterLineGauss.class.getName())`
- `clsApplicableFilters.add(FilterAreaMask.class.getName())`
- `clsApplicableFilters.add(FilterEnhanceContrast.class.getName())`
- `clsApplicableFilters.add(FilterCrossCorrelation.class.getName())`
- `clsApplicableFilters.add(yourFilter.class.getName())`

Then, rebuild the project (by recompiling project in IDE or else).
